# Integrated analysis identifies P4HA2 as a key regulator of STAT1-mediated colorectal cancer progression and a potential biomarker for precision therapy

**DOI:** 10.3389/fonc.2025.1581860

**Published:** 2025-05-08

**Authors:** Qianshi Zhang, Yinan Zhang, Zhiwei Sun, Huanle Wang, Guohang Dai, Yue Meng, Shasha Shi, Shuangyi Ren

**Affiliations:** ^1^ Department of Gastrointestinal Surgery, The Second Affiliated Hospital of Dalian Medical University, Dalian, China; ^2^ Department of Clinical Laboratory, The Second Affiliated Hospital of Dalian Medical University, Dalian, China; ^3^ Department of Ultrasound, The Second Affiliated Hospital of Dalian Medical University, Dalian, China

**Keywords:** P4HA2, colorectal cancer, STAT1, prognostic marker, PD-L1

## Abstract

**Introduction:**

P4HA2 is implicated in regulating tumor microenvironment formation and may play roles in inflammation and tumor immunity. However, its mechanistic involvement in colorectal cancer (CRC) remains largely unexplored.

**Methods:**

We analyzed P4HA2 expression in CRC tissues and correlated it with clinicopathological features. Functional assays (CCK8, wound healing, Transwell) were performed to assess proliferation and migration. Proteomic analysis identified downstream targets, with STAT1/PD-L1 pathway validation.

**Results:**

High P4HA2 expression correlated with advanced T/M stages and served as an independent poor prognostic factor. Functional experiments confirmed P4HA2's role in promoting CRC proliferation and migration. Mechanistically, P4HA2 bound to and downregulated STAT1, subsequently modulating the STAT1/PD-L1 pathway.

**Discussion:**

Our findings reveal P4HA2 promotes CRC progression and suppresses anti-tumor immunity via STAT1/PD-L1 axis regulation. This study uncovers a novel pathogenic mechanism, positioning P4HA2 as a potential therapeutic target in CRC.

## Introduction

Colorectal cancer (CRC) is the most prevalent gastrointestinal malignancy in Southeast Asian countries. In 2020, the age-standardized incidence rate was 19.5/100,000, and the number of new cases was 1,931,590 (1). China has the highest age-standardized incidence rate, accounting for 28.2% of the total (2). Although early detection of colorectal cancer has garnered attention, patients still have to address the disease’s short survival time and unfavorable prognosis due to invasion and distant metastases. Therefore, a key area of study in the clinical research on colorectal cancer is the molecular mechanism of the disease’s onset and progression.

Tumor development and occurrence are the results of pathological alterations involving numerous variables. prolyl 4-hydroxylase subunit alpha 2 (P4HA2) is a component of proline 4-hydroxylase (P4H) and is one of the three P4HA subtypes found in the human body (P4HA1, P4HA2, and P4HA3) (3). P4H is an essential enzyme in the synthesis of collagen and consists of two α subunits that are identical and two β subunits (4). The production of 4-hydroxyproline, which is catalyzed by P4H, is crucial for the proper three-dimensional folding of the freshly synthesized procollagen chain. Collagen is the main component of the extracellular matrix (ECM) (5). As the most abundant component in the tumor microenvironment, collagen can regulate tumor cell behavior and progression. The most prominent fibrous collagen among them is type I collagen, which is highly expressed in a range of cancers and accelerates the development of cancer by encouraging the growth, migration, invasion, and epithelial–mesenchymal transition of tumor cells as well as their resistance to chemotherapy (6, 7). Collagen type IV is a nonfibrous collagen required for the formation of the basement membrane, which promotes tumor cell migration (8). P4HA2 interacts with the aforementioned molecules of the collagen family to regulate proline hydroxylation, which in turn promotes proline metabolism and influences tumor progression. Research has demonstrated that in hypoxic environments, P4HA2 can cause extracellular matrix remodeling (9). Increased expression of P4HA2 has been detected in breast cancer (10), oral squamous cell carcinoma (11), papillary thyroid carcinoma (12), lung adenocarcinoma (13), B lymphoma (14), and glioma (15). Nevertheless, research has not been able to establish the role of P4HA2 in colorectal cancer.

As a transcription factor, cytokine, and signal transduction molecule, STAT1 plays a crucial role in regulating gene expression at both the transcriptional and epigenetic levels, facilitating cell apoptosis, and inhibiting tumor pathogenesis, such as proliferation, differentiation, and drug resistance (16). STAT1, a key molecule in signal transduction within the tumor microenvironment, is intimately associated with inflammatory responses and tumor immune surveillance (17). An increasing body of evidence indicates that the IFN-γ/JAK2/STAT1 pathway is widely regarded as the principal inducer of PD-L1 expression (18, 19). STAT1 serves as a key effector that modulates PD-L1 expression, and activated STAT1 binds to the PD-L1 promoter to upregulate the expression of PD-L1 (20). Studies have also demonstrated that HKDC1 enhances the expression of PD-L1 by binding with STAT1, thereby augmenting the immune escape capacity of tumor cells (21). All the aforementioned studies suggest that STAT1 governs the expression of PD-L1 and participates in the regulation of anti-PD-L1 therapy. Additionally, continuous activation of the IFN-γ/STAT1 pathway and mutations in signaling molecules in tumor cells increase the likelihood of developing resistance to PD-L1 inhibitors (22, 23).

The objective of our study was to explore the mechanism through which P4HA2 promotes CRC cell proliferation and migration *in vitro*. Our data indicate that P4HA2 increases the expression of PD-L1 by binding to and downregulating STAT1 expression, thereby increasing the risk of cancer. In conclusion, this study is intended to offer novel insights into the molecular mechanism by which P4HA2 facilitates CRC progression and anti-PD-L1 resistance.

## Materials and methods

### Bioinformatics analysis

The gene expression dataset GSE87211 (203 colon cancer samples and 160 matched normal controls) was obtained from GEO. Raw data from the Agilent GPL13497 platform were processed using Strawberry Perl for probe-to-gene conversion. Data normalization and correction were performed using BiocManager and Limma packages in R. Survival-associated genes (p < 0.001 by KM/Cox analysis) were identified via the survival package. Independent prognostic analysis (p < 0.001) and clinical correlation analysis (p < 0.05) were subsequently conducted. Differentially expressed genes (DEGs) were defined as |log FC| > 0.5 with adjusted p < 0.05 using Limma, and visualized through heatmaps and volcano plots. Functional enrichment analyses (GO/KEGG, p < 0.05) were performed using ggplot2-based packages. All statistical analyses, including t-tests/Wilcoxon tests for group comparisons and Kaplan-Meier/log-rank tests for survival analysis, were implemented in R.

### Microarray analysis

A total of 3 pairs of P4HA2 stable knockdown and control CRC cell pellets were collected by centrifugation at 1000×g for 5 min. The proteins were extracted and detected by 4D label-free quantitative proteomics technology (Shanghai Applied Protein Technology), which is based on differentially expressed peptides in P4HA2-knockdown cells subjected to liquid chromatography (LC)/mass spectrometry (MS) analysis. The differentially expressed proteins had a fold change ≥1.5 and p < 0.05, and the above analysis was performed by Jingjie Bio.

### Cell culture

Human colorectal cancer cell lines (LoVo, SW480, SW620, HT29, and HCT8) were purchased from Dalian Meilun Biotechnology Co. (Dalian, China). Cell lines were authenticated by STR profiling (ATCC), all cell lines were tested for mycoplasma and found to be mycoplasma free. The human normal intestine epithelial cell line was a gift from the Xiangya Experiment Center (Changsha, China). The LoVo and HCT8 cells were cultured in RPMI 1640 medium (Gibco, California, USA), the SW480 cells were cultured in DMEM (Gibco, California, USA), and the SW620 cells were cultured in Leibovitz’s L-15 medium (Gibco, California, USA) supplemented with 10% fetal bovine serum (Gibco, California, USA) and 100 U/ml penicillin–streptomycin-amphotericin B solution (Procell, Wuhan, China) at 37°C in a humidified incubator with 5% CO_2_.

### siRNA treatment and lentivirus transduction

In this study, the pG-LV5 lentiviral vector (LV5) and pG-LV3 lentiviral vector (LV3) (GenePharma Co. Ltd., Shanghai, China), depending on the P4HA2 coding sequence (CDS) or the shP4HA2 sequence, were employed to construct LV5-P4HA2 and LV3-shP4HA2, respectively. The lentivirus infection was performed in accordance with the manufacturer’s protocol. After infection for 24 h, the medium was replaced with fresh medium. Forty-eight hours after infection, puromycin (Beijing Solaibao Technology Co. Ltd., China) was added to the culture medium to select the infected cells. The sequences and NCBI identifiers are available in **Supplementary Table S1**.

### CCK8

The proliferation of the cells was tested via a Cell Counting Kit 8 (MCE; Monmouth Junction, NJ, USA) according to the manufacturer’s instructions. An enzyme-labeling instrument was then used to measure the OD value at a wavelength of 450 nm at Days 1, 2, 3, and 4.

### Transwell

A Transwell assay (24-well, Corning, NY, USA) was used to measure the migratory ability of the cells. Briefly, 3 × 10^5^ cells (200 µl) in serum-free medium were added to the upper chamber for the migratory assay. The cells were incubated for 24 h, fixed, and stained with 0.5% crystal violet solution. The average number of cells was calculated from five different microscopic fields.

### Wound healing assay

For the process of wound healing, 3 × 10^5^ cells were seeded in 12-well plates and incubated overnight. A vertical scratch was produced via a 10 μL pipette tip, which was subsequently washed twice and cultured in serum-free medium. The scratch width, a migration index, was observed and photographed under a microscope (Leica, Germany) at 0 and 24 hours and was measured via ImageJ software.

### Immunohistochemistry

The tumor specimens from 34 patients were suitable for IHC analyses. With approval and support from the ethics committee of the Second Affiliated Hospital of Dalian Medical University. All experiments were performed in accordance with relevant guidelines and regulations. Informed consent was obtained from all patients.Colon cancer tissues were fixed in 10% formalin, embedded in paraffin, and serially cut into 4 μm thick sections. HE staining and immunostaining were performed on 4 μm sections. For HE staining, the tissues slices were paraffin-embedded, dewaxed, rehydrated, and stained with HE according to the manufacturer’s protocol for the HE staining kit (Beyotime, Jiangsu, China). For immunostaining, the slices were stained according to the manufacturer’s protocol for the immunostaining kit (ZSGB-Bio, China). The tissue slides were deparaffinized with dimethylbenzene, hydrated with gradient ethanol, subjected to antigen retrieval, and blocked with normal goat serum. Endogenous peroxidase (HRP) activity was blocked by treating the sections with 3% hydrogen peroxide. The sections were incubated overnight with a rabbit antibody against P4HA2 (1:200 dilution). After rinsing with phosphate-buffered saline (PBS), a horseradish peroxidase (HRP)-conjugated secondary antibody was applied, and the samples were stained with a diaminobenzidine (DAB) kit (ZSGB-Bio, China). After immunohistochemistry, the tissue sections were counterstained with hematoxylin. Slides were scanned via a Leica microscope. To evaluate immunohistochemical staining for P4HA2, the staining intensity was scored as 0 (no staining), 1 (weak staining), 2 (intermediate staining), or 3 (strong staining), and the staining area was scored as 0 (0–10% positive cells), 1 (11–33% positive cells), 2 (31–50% positive cells), 3 (51–70% positive cells) or 4 (71–100% positive cells). The immunoreactivity scores (IHC scores) were determined by multiplying the staining score by the percentage score to obtain a maximum of 12.

### Quantitative real-time PCR

Total RNA was extracted from cultured cells using a SteadyPure Universal RNA Extraction Kit (Accurate Biology, Changsha, China), and the raw RNA concentration was measured via a NanoDrop 2000 (Thermo Fisher Scientific, Waltham, MA, USA). Two micrograms of total RNA per sample was reverse transcribed into cDNA by using the FastKing One-Step RT–PCR Kit (TIANGEN). The primers used are listed in **Supplementary Table S2**. q-PCR analyses were performed with SuperReal PreMix Plus (TIANGEN) on the QuantStudio™ 5 Real-Time System (Invitrogen, Carlsbad, CA). GAPDH was used as the internal reference, and the 2-ΔΔCt method was used to calculate the relative expression of the target genes.

### Western

Protein was extracted from cultured cells using a total protein extraction kit (KeyGen, Jiangsu, China), and the protein concentration was measured via a BCA protein assay kit (Seven Biotech). The proteins were electrophoresed on 10% SDS polyacrylamide gels (Shanghai Epizyme Biomedical Technology Co., Ltd.) and transferred to nitrocellulose membranes (Millipore) using NcmSafe Blue Protein Stain (NCM Biotech). The membranes were then incubated with the corresponding primary antibodies and secondary antibodies. β-actin (1:500, ZSGB-BIO) was used as a loading control. Anti-P4HA2 (1:500, ab211527) and anti-STAT1 (1:500, ab234400) were purchased from Abcam (Cambridge, MA, USA), and anti-rabbit IgG (H+L) DyLight™ 680 and anti-mouse IgG (H+L) DyLight™ 680 cross-adsorbed secondary antibodies (1:10000, Invitrogen, Carlsbad, CA) were used. The signals were detected with a near-IR fluorescence scanner (Odyssey Imaging System, LICOR).

### Coimmunoprecipitation

Immunoprecipitation assays were performed using the Crosslink Magnetic IP/Co-IP Kit (Thermo Scientific) in HCT8 cells according to the manufacturer’s instructions. The cells were solubilized with immunoprecipitation buffer, and equal amounts of protein were incubated with specific antibodies immobilized onto protein A/G magnetic beads overnight at 4°C with gentle rotation.

Protein G magnetic beads were used for Co-IP, and IP was performed with 5 μg of P4HA2 polyclonal antibody (Abcam, ab70887) and rabbit IgG (as a negative control; Abcam, ab171870). The beads were washed extensively with immunoprecipitation lysis buffer, resuspended, and eluted. Protein samples were subjected to SDS–PAGE followed by Western blot analysis. Western blots were performed individually using the following antibodies: anti-P4HA2 (1:500, Abcam, ab211527) and anti-STAT1 (1:500, Abcam, ab70887).

### Protein 3D structure prediction

Based on the protein ID predicted by quantitative proteomics, we chose the protein structures of P4HA2 (ID: 6EVM) and STAT1 (ID: 1YVL) from the PDB database for further analysis. For protein–protein docking, ClusPro, an online protein docking server, was used with its default parameters. ClusPro then generated multiple interaction models ranked in the order of stability. Interaction model structures at the first position of the ranking were visualized and rendered via PyMOL 2.0.5 (24–27).

### Statistical analysis

All the experiments were repeated at least three times, and the data are presented as the means ± SDs. The statistical analyses were performed via GraphPad Prism 8.0. The graphs were analyzed by either ANOVA (multiple groups) or t tests (two groups). A P value of 0.05 or less was considered statistically significant.

## Results

### P4HA2 might be a specific molecular marker for colorectal cancer

**Figure 1:**
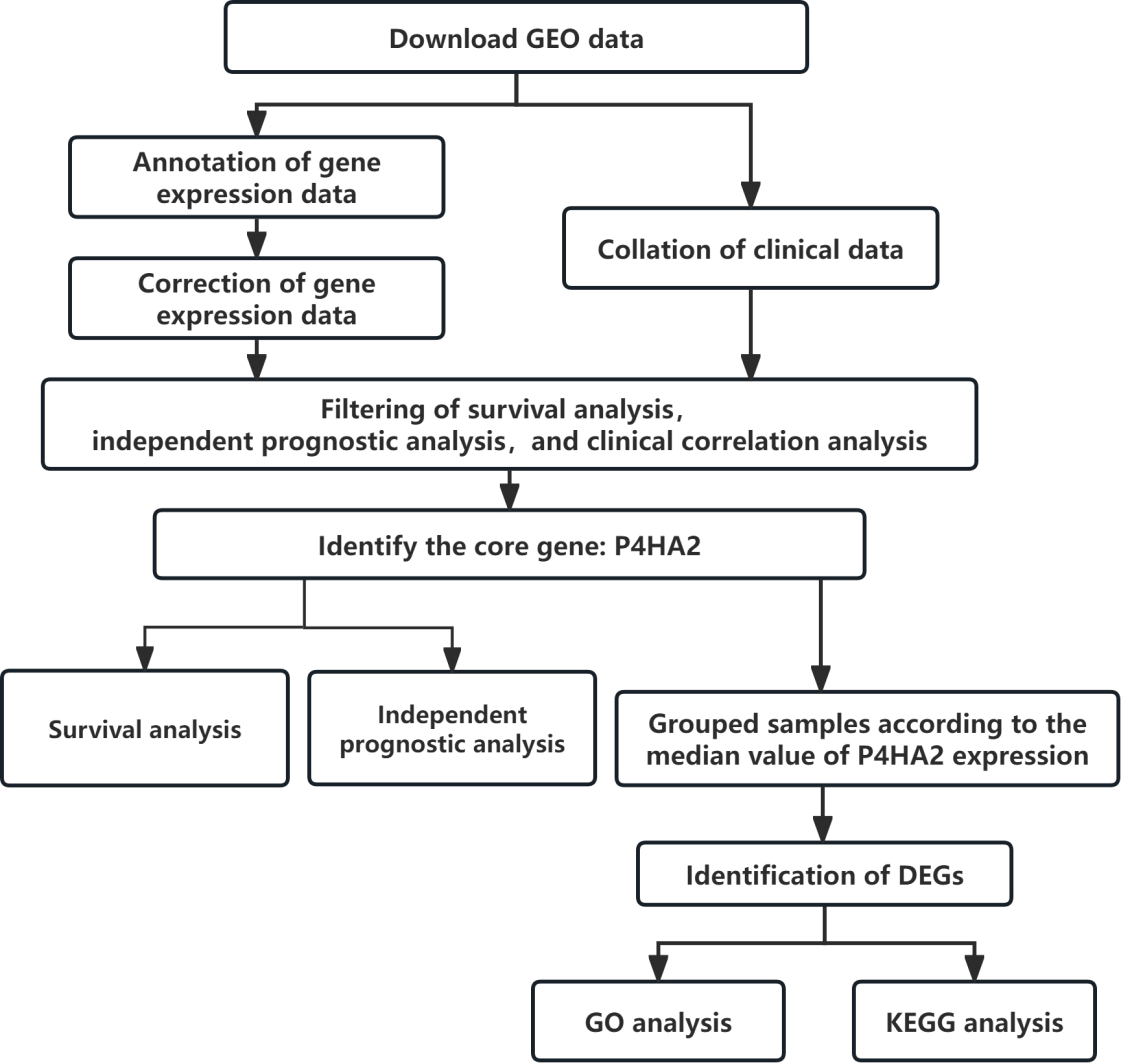
Flow chart of the identification and potential mechanisms of the P4HA2 core gene in colorectal cancer.

This study utilized the colorectal cancer microarray dataset GSE87211 from the GEO database, comprising 203 tumor tissue samples and 160 paired adjacent normal mucosal tissues. The gene expression data were annotated and corrected, and the clinical data of the samples were organized. Through sequential survival analysis, independent prognosis analysis, and clinical correlation analysis, the genes were progressively filtered down to 283, 148, and 26 core genes, respectively. The genes most significantly related to clinical indicators and with the highest expression levels were selected as core genes (**Table 1**), and P4HA2 is the most prominent core gene. DEGs between the high- and low-expression groups (based on the median expression values) were identified, followed by biological function enrichment analysis (**Figure 1**).

**Table 1:** Genes strongly correlated with clinical indicators and their average expression.

Gene id	Rct	bT	bN	bM	aT	aN	aM	SigNum	AveExpr
EVC2	0.405256828	0.409690082	0.029040605	0.002961412	0.623687061	0.122548761	0.000273199	2	3.863224701
GPSM2	0.234880245	0.350394458	0.374377019	0.006230619	0.033212312	0.059616062	0.003548324	2	3.863224701
HSD17B7	0.288288923	0.944666602	0.107424945	0.001573561	0.97212483	0.354653145	0.000362046	2	8.854864765
NFATC1	0.463565955	0.787590236	0.275683927	0.001350247	0.92240259	0.52542954	0.003207597	2	8.757857958
*P4HA2*	*0.848460619*	*0.971531265*	*0.007668288*	*0.613463441*	*0.297202614*	*0.008124974*	*0.280567116*	***2***	*12.49963284*
ACP1	0.716336033	0.712908439	0.505709827	0.035186309	0.839290797	0.048044958	0.003153637	1	–
ATP6V1E2	0.788211942	0.354888259	0.467116383	0.076328663	0.086977742	0.009281238	0.054816678	1	–
BRCA1	0.006846089	0.721635934	0.415619067	0.262901202	0.701874237	0.102954831	0.298411175	1	–
C17orf42	0.741707144	0.111797953	0.640806539	0.036755046	0.692619927	0.11289442	0.006082074	1	–
C1GALT1C1	0.181889587	0.978556871	0.256416397	0.00991343	0.544524986	0.901904324	0.012225575	1	–
DIEXF	0.287746248	0.075437149	0.068965187	0.375510544	0.447770266	0.005916737	0.140601008	1	–
DNAJC17	0.727952187	0.639611873	0.651873711	0.011071159	0.721506128	0.543651069	0.008513727	1	–
EXT1	0.770992497	0.875442658	0.866478216	0.521512398	0.474767282	0.004905725	0.088480096	1	–
GPR173	0.5852544	0.432444819	0.562817661	0.078308935	0.008182499	0.176038547	0.44396262	1	–
GRID1	0.698736447	0.160517531	0.689659122	0.048802405	0.160174575	0.001986545	0.03216098	1	–
HELLS	0.009134673	0.957054529	0.440106798	0.517681438	0.83323513	0.451118979	0.439312749	1	–
IL22RA1	0.907120753	0.251640034	0.093791028	0.022059533	0.008238967	0.435134313	0.010612549	1	–
LMO4	0.02990754	0.062713136	0.474011448	0.131174567	0.56513597	0.000475501	0.0782475	1	–
MAS1L	0.643253028	0.164050552	0.582842661	0.489417954	0.001584709	0.14877001	0.761835046	1	–
OPN1SW	0.416593266	0.072984227	0.298512735	0.738529276	0.007950931	0.458350727	0.576082761	1	–
OR13C4	0.132938477	0.546955128	0.63380438	0.079822247	0.422513144	0.003871501	0.182705521	1	–
OR2A7	0.009220277	0.844251607	0.050470011	0.097480514	0.079128408	0.134804063	0.297196682	1	–
OR2W1	0.561681922	0.680749452	0.369125105	0.346027393	0.007741893	0.66830438	0.092965854	1	–
OR4S1	0.141744718	0.057789558	0.662004576	0.751944117	0.007959741	0.572051237	0.941682644	1	–
PBOV1	0.634815713	0.056762667	0.751507723	0.586761587	0.008693355	0.583295115	0.606763011	1	–

**Figure 2:**
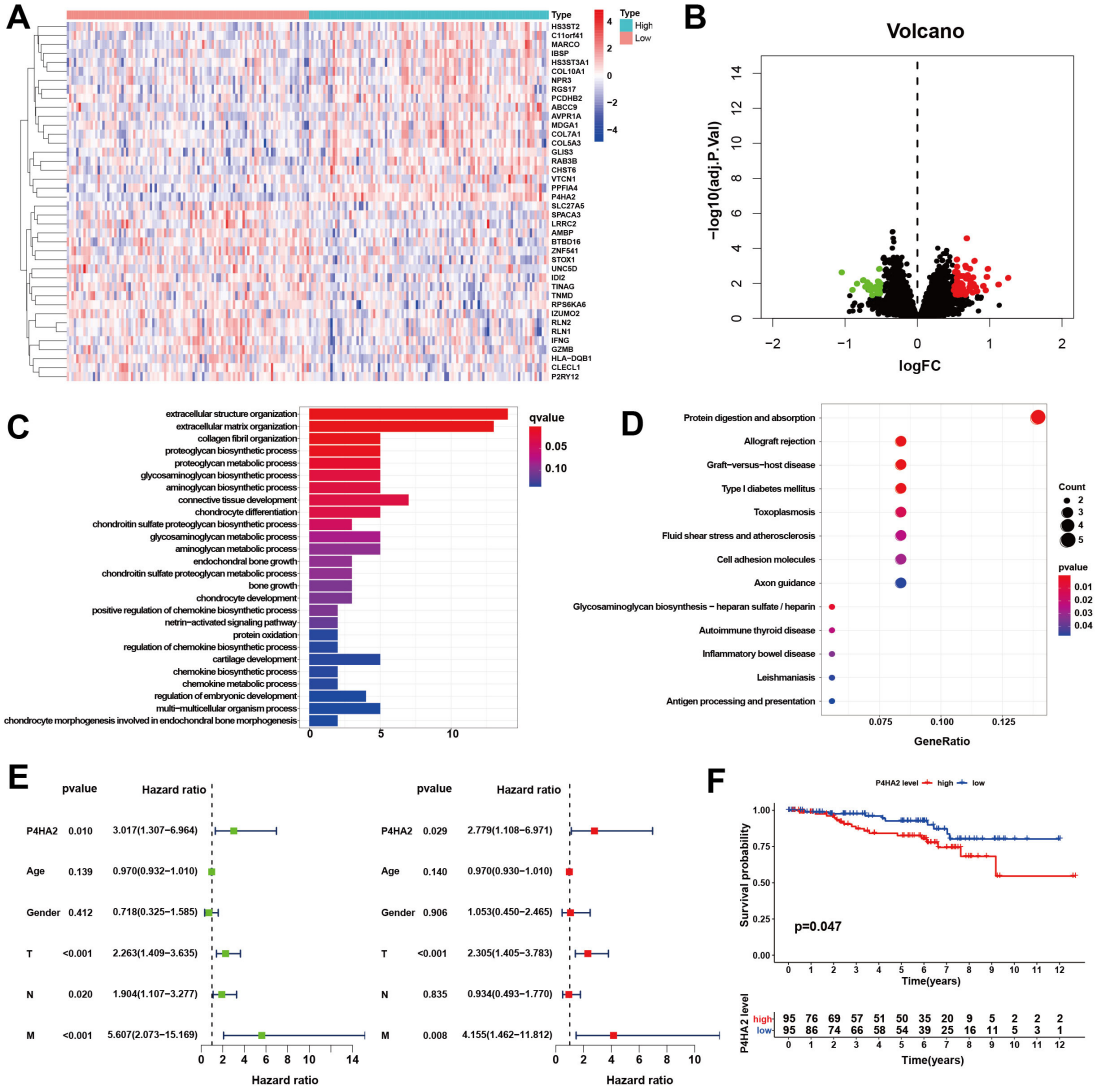
Cluster analysis of differentially expressed genes. **(A)** Heatmap. **(B)** Volcano map. The low-expression samples are shown in blue, the high-expression samples are shown in red, and the middle-expression samples are shown in white. Volcano plots were constructed using |log2(FC)| > 0.5 and adj P < 0.05, with the green dots indicating the downregulated differentially expressed genes and the red dots indicating the upregulated ones. **(C, D)** Biological functions and signaling pathways enriched by the DEGs. **(C)** GO analysis, **(D)** KEGG pathway analysis. **(E)** Forest plot of the hazard ratios evaluating P4HA2 expression as an independent predictor. Univariate analysis (L), including age, sex, tumor TNM stage, and multivariate Cox regression analysis (R), was performed. **(F)** Kaplan–Meier curve of OS in patients with colorectal cancer. Patients were divided into two groups according to the median P4HA2 expression level: the high-expression group (red) and the low-expression group (blue).

From the GSE87211 dataset, the remaining genes were categorized into high- and low-expression groups according to the median expression value of P4HA2. DEGs with statistical significance were defined as those with a |log2(FC)| > 0.5 and P < 0.05, resulting in 86 DEGs: 62 upregulated and 24 downregulated (**Supplementary Table S3**). These findings were visualized via heatmaps and volcano plots (**Figures 2A, B**).

The 86 DEGs were subjected to functional analysis. Biological processes (BP) included extracellular structural organization, collagen fibril organization, and proteoglycan metabolism. Cellular components (CCs) included the extracellular matrix and collagen structures. Molecular functions (MFs) included structural components of the extracellular matrix. The key KEGG pathways involved antigen processing and presentation, cell adhesion molecules, and glycosaminoglycan biosynthesis (**Figures 2C, D**). To determine whether P4HA2 expression is independent of other clinical characteristics, univariate and multivariate Cox regression analyses were conducted. Univariate analysis indicated that P4HA2 expression, tumor T stage, and M stage had significant impacts on patient prognosis. Multivariate analysis revealed P4HA2 expression was an independent prognostic factor (P = 0.029, hazard ratio (HR): 2.779, 95% CI: 1.108–6.971) for high risk (**Figure 2E**).

The prognostic value of P4HA2 expression was further assessed by comparing the overall survival (OS) between the high- and low-expression groups. The results demonstrated that the OS of the low-expression group was significantly better than that of the high-expression group (P = 0.047), as shown in **Figure 2F**.

### P4HA2 is increased in CRC and correlates with CRC progression

**Figure 3:**
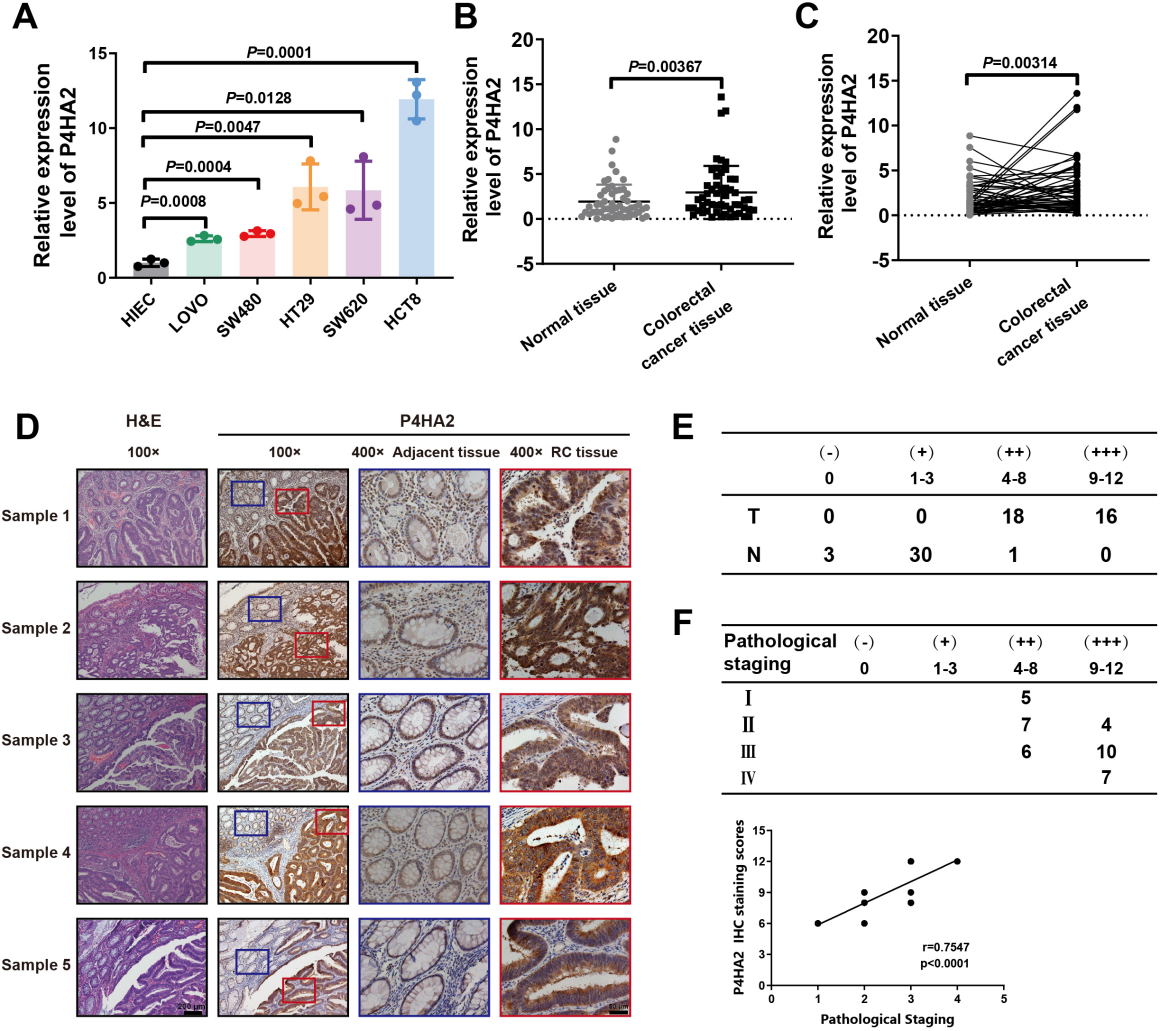
P4HA2 is increased in CRC and is correlated with CRC progression. **(A)** The expression of P4HA2 was validated in both colorectal cancer cell lines and the HIEC cell line, with the relative expression of P4HA2 in these cell lines determined by qRT–PCR. **(B, C)** P4HA2 expression was confirmed in colorectal cancer tissues. The expression levels of P4HA2 were greater in colorectal cancer tissues than in adjacent normal tissues. **(D)** Representative images of IHC staining. **(E)** The detailed scoring criteria and rating criteria are shown. **(F)** The IHC score and pathological stage were positively correlated.

Analysis of the data from the TCGA database suggested that high P4HA2 expression in colorectal cancer specimens predicts a poor outcome in patients with colorectal cancer. To clarify the role of P4HA2 in CRC, we further detected P4HA2 expression in five colon cancer cell lines (HCT8, SW620, HT29, SW480, and LoVo cells) and one normal human intestinal epithelial cell line (HIEC cells). The results revealed that P4HA2 was notably upregulated in the colorectal cancer cell lines compared with the normal HIEC cell line (**Figure 3A**).

Furthermore, we examined P4HA2 expression levels in 54 pairs of CRC tissues and matched nontumor tissues, which also revealed that P4HA2 was overexpressed in human colorectal cancer tissues compared with matched nontumor tissues (**Figures 3B, C**). Then, immunohistochemical analysis of 34 matched tumor and nontumor tissues was carried out to comprehensively characterize P4HA2 expression in colorectal cancer tissues, which confirmed the increased expression of P4HA2 in cancer tissues (**Figures 3D, E**). Moreover, a significant difference in P4HA2 expression levels between stages III/IV and stages I/II was observed (**Figure 3F**). Collectively, the data above suggest that P4HA2 may act as an oncogene in colorectal cancer tumorigenesis and progression.

### P4HA2 positively regulates CRC cell proliferation and migration *in vitro*

**Figure 4:**
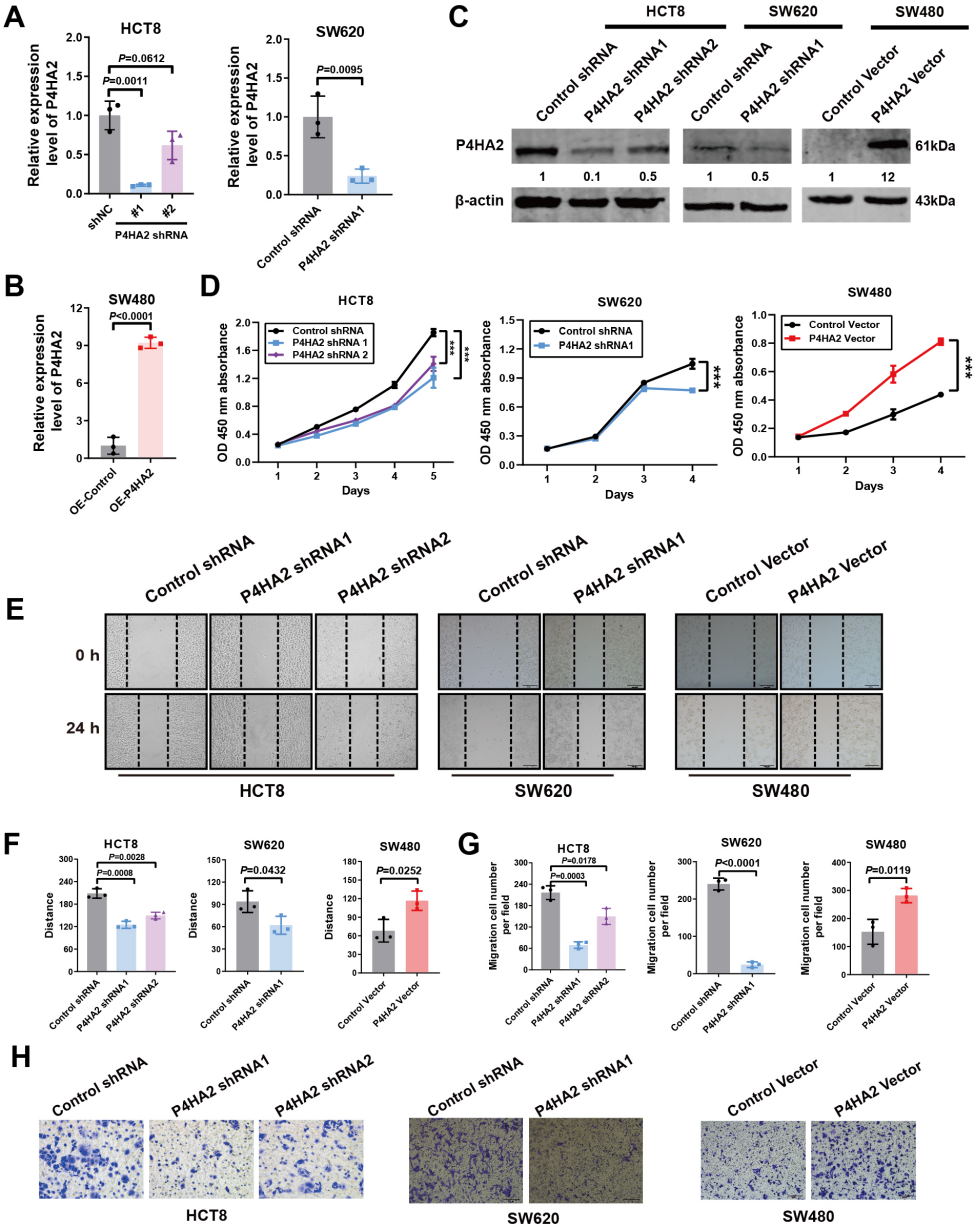
P4HA2 positively regulates CRC cell proliferation and migration *in vitro*. **(A-C)** Effects of P4HA2 knockdown and overexpression. qRT–PCR and Western blotting revealed that the pLKO.1-shP4HA2 or pLenti-CMV-P4HA2 lentiviral virus vectors had a knockdown or overexpression effect on P4HA2, respectively. **(D)** The proliferation of CRC cells treated with P4HA2 was determined via a CCK8 assay. **(E, F)** Wound healing assay revealed that P4HA2 promoted the migration of CRC cell lines. **(G, H)** The effect of P4HA2 on CRC cell migration was examined via Transwell migration assay, and the number of migrated cells was determined via ImageJ software.

To assess the contribution of P4HA2 to cell growth and metabolism, we ectopically expressed P4HA2 and knocked down P4HA2 in CRC cells via two shRNAs and validated the knockdown efficiency via qRT–PCR and Western blotting. We used two P4HA2 target shRNA sequences to generate stable CRC lines with different efficiencies of P4HA2 depletion (~15% knockdown with shRNA_1 and ~60% knockdown with shRNA_2) (**Figures 4A, C**). Cells with stable P4HA2 overexpression were generated via lentivirus infection, and P4HA2 overexpression was confirmed via qRT–PCR and Western blotting, demonstrating P4HA2 overexpression by more than nine-fold (**Figures 4B, C**).

To investigate the biological function of P4HA2 in CRC progression, we conducted a CCK8 assay and revealed a significant difference in P4HA2 knockdown or over-expression between the control groups (**Figure 4D**). Notably, HCT8 and SW620 cells with P4HA2 knockdown presented a significantly low proliferation rate. In contrast, the opposite result was observed with overexpression of P4HA2 in SW480 cells, which indicated a growth-promoting effect of P4HA2 in CRC cells. The wound healing assay results indicated that P4HA2 promoted the migration of CRC cells (**Figures 4E, F**). Moreover, this promoting function was also substantiated by the transwell assay results (**Figures 4G, H**). These *in vitro* results indicated that P4HA2 promotes CRC cell proliferation and migration.

### P4HA2 downregulates STAT1 expression

**Figure 5:**
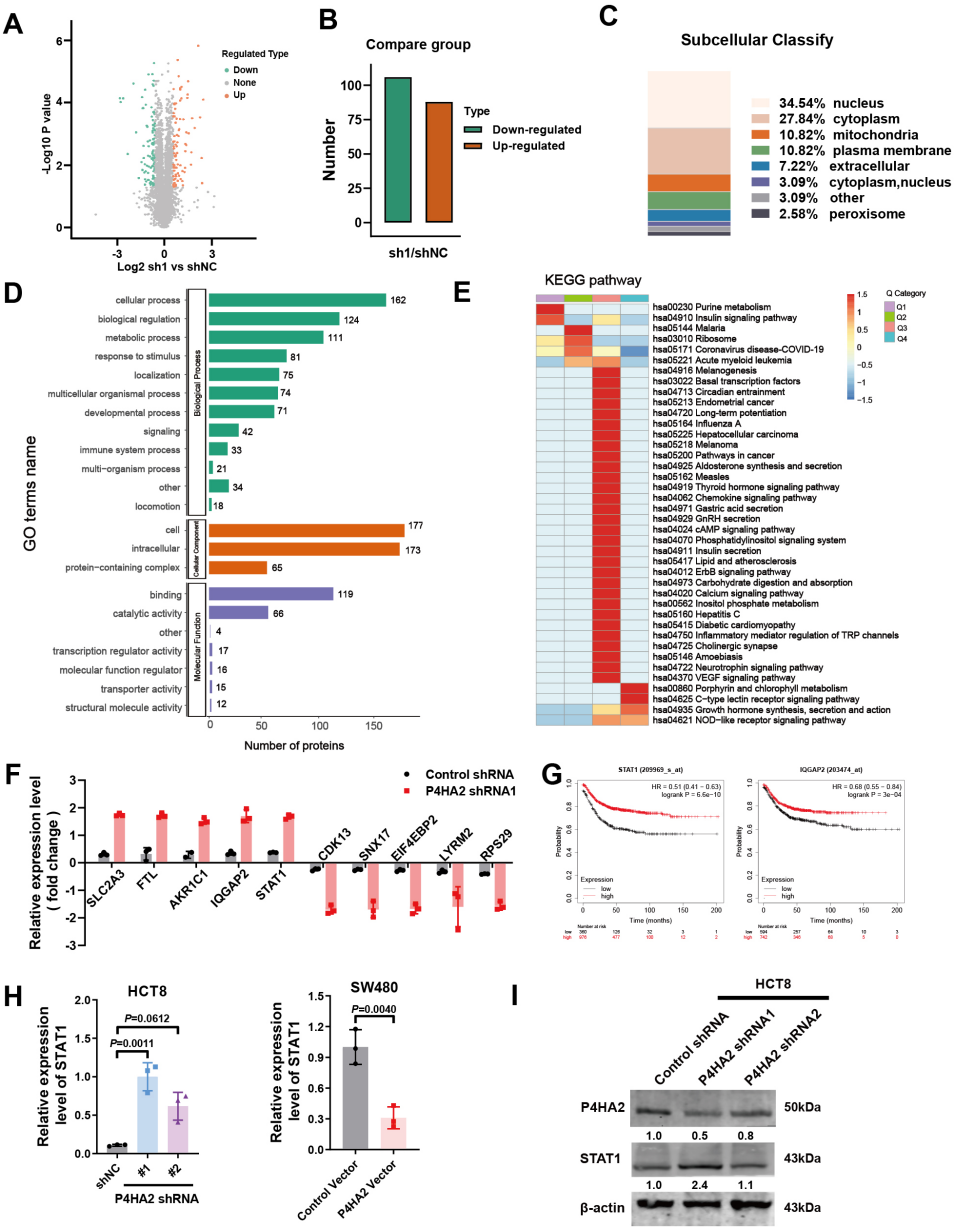
Quantitative analysis of differentially expressed proteins in P4HA2-knockdown CRC cells. **(A)** Volcano plot was generated to visualize differences in protein expression. **(B)** The number of differentially expressed proteins in P4HA2-knockdown CRC cells, 88 of which were upregulated and 106 of which were downregulated. **(C)** Quantitative analysis of the subcellular localization of the differentially expressed proteins. **(D)** GO enrichment analyses of the differentially expressed proteins in M. separata. **(E)** KEGG enrichment analyses of the differentially expressed proteins in M. separata. **(F)** The top 5 upregulated and downregulated proteins are presented. **(G)** Kaplan–Meier (KM) survival analysis of STAT1 and IQGAP2. **(H, I)** The expression of STAT1 mRNA and protein induced by P4HA2 in CRC cell lines was measured by qRT–PCR and Western blotting.

A 4D label-free quantitative proteomics array revealed the molecules involved in P4HA2 knockdown in CRC cells, and a volcano plot was generated to visualize the differences in protein expression (**Figure 5A**). Among these proteins, 88 were upregulated, and 106 were downregulated (**Figure 5B**). The percentages of differentially expressed proteins subcellular localized to either the nucleus, cytoplasm, mitochondria, or plasma membrane were quantified and displayed as bar graphs. The subcellular localization of differentially expressed proteins may reflect their biological functions (**Figure 5C**).

We next performed Gene Ontology and KEGG pathway analyses, which revealed that the differentially expressed proteins were involved in multiple pathways related to tumor proteins regulated by P4HA2 (**Figures 5D, E**). The GO assignments of the three categories are shown in **Figure 5D**. Gene ontology analysis revealed GO enrichment terms, such as “cellular process”, “biological regulation”, and “metabolic process” in the biological process category and “cell and intracellular” in the cellular component category. In terms of molecular function, the category “binding”, which included 119 uni proteins, was the largest, followed by “catalytic activity”. The differentially expressed proteins related to these pathways were subsequently associated with regulatory processes on the basis of the following KEGG pathways: pathways in cancer and inflammatory mediator regulation of TRP channels.

Through a quantitative proteomics array, differentially expressed proteins were defined as those with an adjusted p value (p-adjust) < 0.05 and a |log2-fold change (FC) | >1.5. We identified 191 differentially expressed proteins after P4HA2 knockdown in CRC cells (**Supplementary Table S4**), and the top 5 upregulated and downregulated proteins are presented in **Figure 5F**. To identify the key proteins related to the overall survival of CRC patients, we performed Kaplan–Meier (KM) survival analysis of the top 5 upregulated and downregulated proteins (**Figure 5G**, **Supplementary Figure S1A**). Finally, a total of 2 downregulated proteins, STAT1 and IQGAP2, with high prediction efficiency in the survival analysis were selected as the candidate predictors. STAT1 and IQGAP2, which are tumor suppressor genes, were upregulated after P4HA2 knockdown. A number of studies have revealed the tumor-suppressing properties of STAT1 in various cancer cells (28, 29), which is consistent with the CRC data collected from the TCGA database and indicated better survival (**Figure 5H**). To validate the proteomics array results, we validated these changes in expression via qPCR. The levels of IQGAP2 did not significantly change after P4HA2 treatment (**Supplementary Figure S1B**), and STAT1 was the most significantly upregulated gene. Western blot analysis revealed an increase in STAT1 expression in shP4HA2-expressing CRC cells (**Figures 5H, I**). These findings suggest that changes in the STAT1 protein are closely related to P4HA2 in CRC.

### STAT1 downregulation by P4HA2 is involved in CRC adhesion and migration

**Figure 6:**
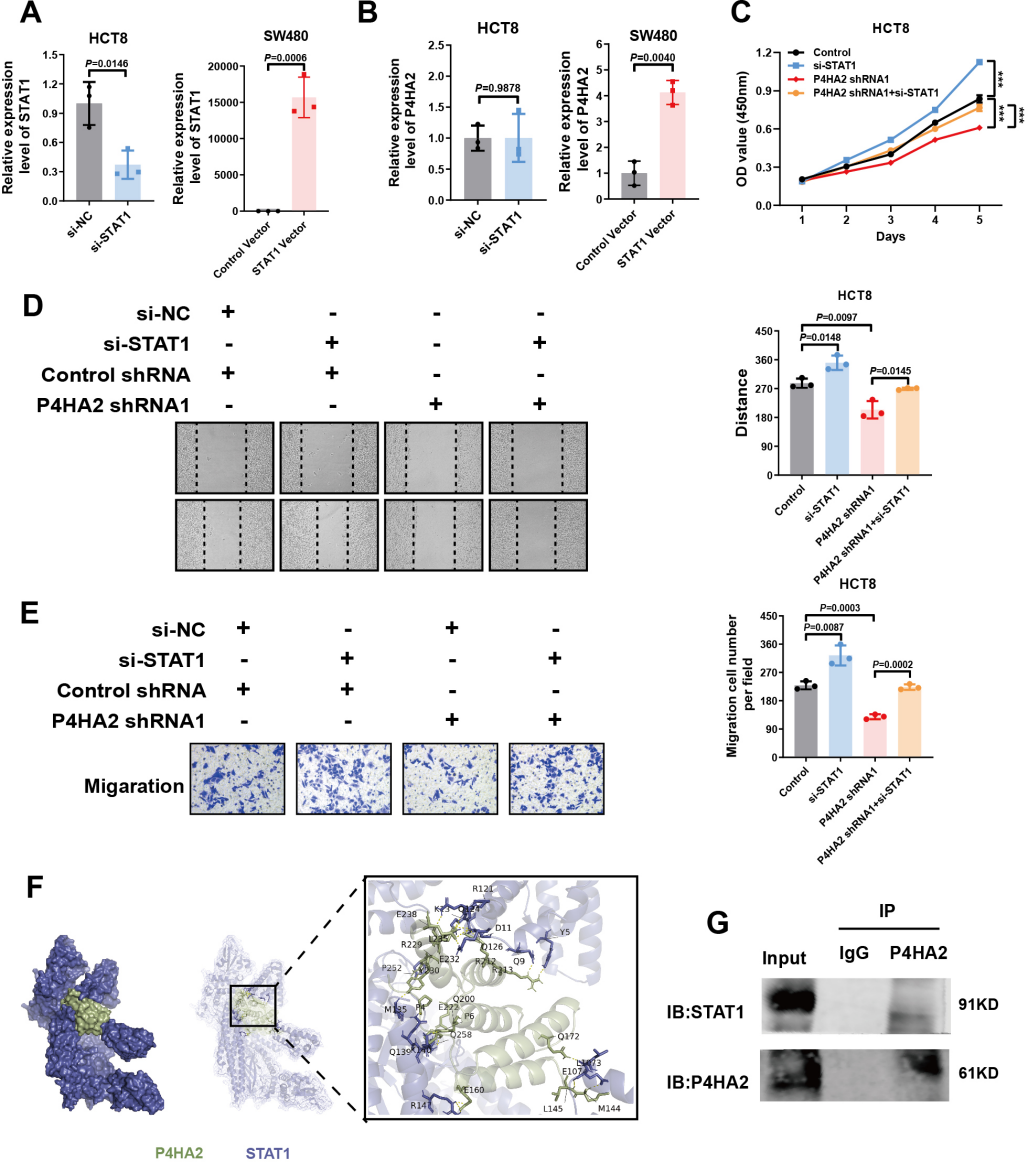
STAT1 downregulation by P4HA2 is involved in CRC adhesion and migration. **(A)** QPCR confirmed the STAT1 expression level upon knockdown or overexpression in CRC cells. **(B)** QPCR was used to detect P4HA2 expression levels after STAT1 knockdown or overexpression in CRC cells. **(C)** CCK8 experiments revealed that STAT1 knockdown promoted the proliferation of HCT8 cells after P4HA2 knockdown. **(D, E)** Wound healing and Transwell migration assays revealed that STAT1 knockdown promoted P4HA2-knockdown CRC cell migration. **(F)** ClusPro was used to predict the interaction between P4HA2 and STAT1. **(G)** Coimmunoprecipitation (Co-IP) experiments confirmed direct binding between P4HA2 and STAT1.

To understand the regulatory mechanisms between P4HA2 and STAT1, we also detected an interaction between P4HA2 and STAT1 in cells. The levels of STAT1 were significantly changed after P4HA2 treatment. The qPCR assay results illustrated that P4HA2 expression was regulated after STAT1 knockdown. Further, whereas no significant differences were observed in HCT8 cells, differences in P4HA2 expression after STAT1 overexpression was observed in SW480 cells (**Figures 6A, B**). In conclusion, the downregulation of STAT1 by P4HA2 promotes CRC progression.

To determine the involvement of P4HA2-regulated STAT1 in CRC tumorigenesis, HCT8 cells were divided into four groups for *in vitro* experiments: the control shRNA + si-NC, control shRNA + si-STAT1, P4HA2 shRNA + si-NC, and P4HA2 shRNA + si-STAT1 groups. We performed CCK8, wound healing, and Transwell assays. Our *in vitro* studies suggest that P4HA2 knockdown inhibits CRC cell proliferation and migration and that this effect can be inhibited when STAT1 is simultaneously silenced (**Figures 6C–E**). The above evidence highlights the pivotal role of STAT1 in P4HA2-induced tumorigenesis of CRC cells. Taken together, these results indicate that the downregulation of P4HA2 could inhibit the proliferation and migration abilities of CRC cells by increasing the expression of STAT1.

Given the underlying GO category of molecular function binding mechanisms, ClusPro was used to predict the possible interaction between P4HA2 and STAT1. The potential binding regions of P4HA2 with STAT1 according to the protein prediction analysis illustrated in **Figure 6F** suggested that P4HA2 could bind to STAT1, which was confirmed by co-IP experiments. For the co-IP experiments, P4HA2 antibodies were used to pull down the STAT1 protein, which was subsequently used to determine the direct binding between P4HA2 and STAT1. We propose that P4HA2 promotes the proliferation and migration of cancer cells by binding to and inhibiting the function of the tumor suppressor protein STAT1.

### P4HA2 regulation enhances PD-L1 expression *in vitro*

To explore the mechanisms underlying this inhibitory effect of P4HA2 on STAT1, we investigated signaling pathways significantly associated with cancer via STAT1. STAT1, a key molecule of signal transduction in the tumor microenvironment, is closely related to the inflammatory response and tumor immune surveillance. Increasing evidence shows that the IFN-γ/JAK2/STAT1 pathway is widely regarded as the main inducer of PD-L1 expression (18, 19). In addition, KEGG pathway analyses of the differentially expressed proteins suggested that tumor-intrinsic oncogenic signals related to the inflammatory mediator regulation of TRP channels may mediate cancer immune evasion and resistance to immunotherapy.

**Figure 7:**
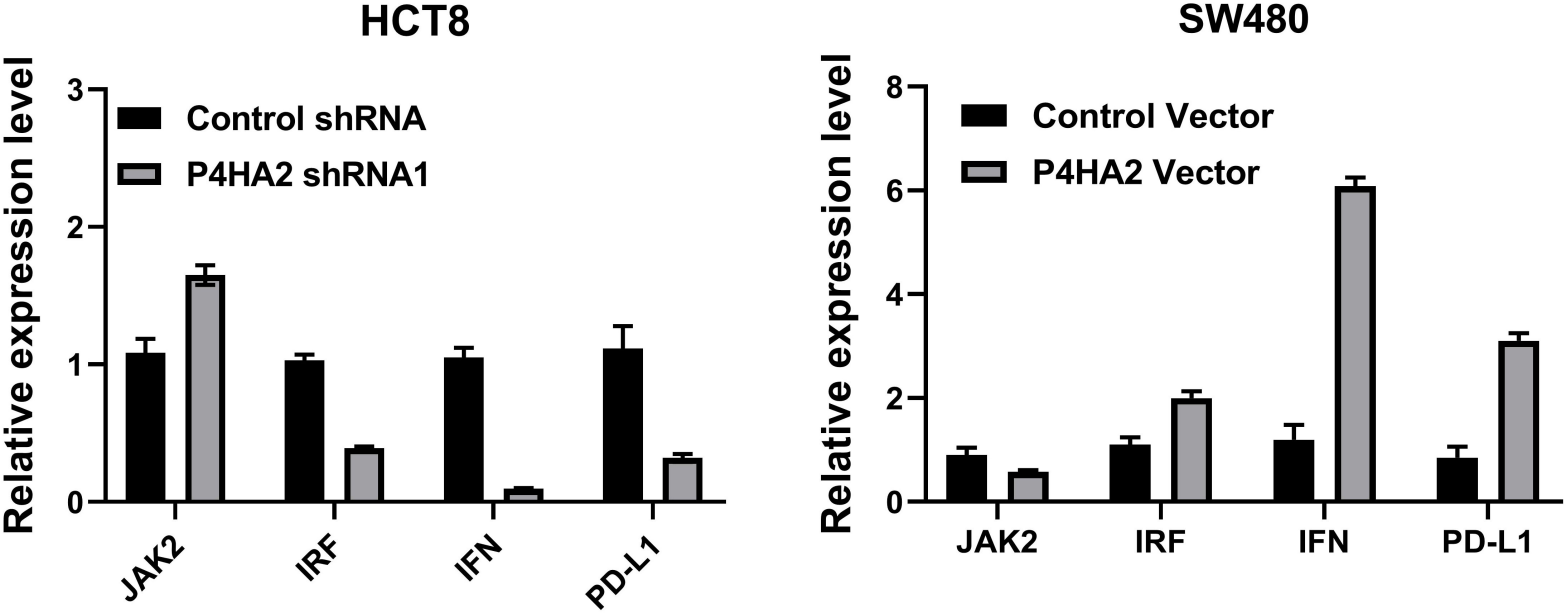
The levels of JAK2, IFN, and PD-L1 were significantly changed after P4HA2 knockdown or overexpression.

To understand the regulatory mechanisms between STAT1 and IFN1/PD-L1, the levels of JAK2, IFN-γ, IRF, and PD-L1 were analyzed (**Figure 7**). We studied the effects of P4HA2 on PD-L1 mRNA levels in CRC cells, and the levels of JAK2, IFN, IRF, and PD-L1 were significantly changed after P4HA2 knockdown (**Figure 7**).

Therefore, PD-L1 expression is regulated by the JAK-STAT1 signaling axis in CRC cells, and the qPCR results suggest that P4HA2 regulates the PD-L1 axis and that PD-L1 is a STAT1 transcriptional target regulated by P4HA2 to facilitate eventual immune evasion.

## Discussion

Colorectal cancer is a relatively common malignant tumor. Its occurrence and development are complex processes that involve variations in multiple genes as well as the participation of expression regulation signaling pathways. Mutations or abnormal expression of genes such as APC (30), KRAS (31), TP53 (32), BRAF (33), and PIK3CA (34) have been shown to disrupt cell growth and differentiation, promoting the occurrence and progression of colorectal cancer. More core genes have been studied to better understand the mechanism of colorectal cancer occurrence and progression, as well as to provide a theoretical foundation for its diagnosis, treatment, and prognosis. In this study, the core gene P4HA2 was identified more accurately via survival analysis filtering, independent prognostic analysis filtering, and clinical correlation filtering of the gene expression data of the GSE87211 dataset. Its high expression is an independent risk factor that influences patient prognosis. P4HA2 is an active subunit that catalyzes the conversion of proline residues in procollagen to 4-hydroxyproline. It is used to catalyze the hydroxylation of proline residues in procollagen to produce 4-hydroxyproline, which is one of the key enzymes for the posttranslational modification of collagen. P4HA2 is involved in several biological processes, including cell signal transduction, angiogenesis, cell proliferation, and apoptosis (15, 35, 36). Several studies have confirmed that P4HA2 is involved in the formation and progression of tumors. In breast cancer, the level of P4HA2 mRNA is significantly greater than that in normal breast tissue, which is associated with a poor clinical prognosis (37). Functionally, HBx-elevated P4HA2 increased collagen deposition in the liver both *in vivo* and *in vitro*, contributing to liver fibrosis and cancer progression (38). In addition, Zhang T et al. confirmed that the PCGEM1/miR-129-5p/P4HA2 axis plays a crucial role in the metastasis and invasion of gastric cancer, indicating that it can be used as a potential biomarker for diagnosis and treatment (39).

To further explore the role of P4HA2 in colorectal cancer, the authors detected the expression level of P4HA2 in colorectal cancer cell lines and tissue samples via qRT–PCR and immunohistochemistry. The findings revealed that P4HA2 was highly expressed in colorectal cancer cells and tissues, which was consistent with the results of the bioinformatics analysis. The author’s analysis of the correlation between abnormally high expression of P4HA2 in colorectal cancer tissues and clinicopathological stages revealed that high P4HA2 expression was positively correlated with colorectal cancer development and was closely related to colorectal cancer lymph node metastasis. These results indicate that P4HA2 is closely related to tumor progression and advanced tumor stage. Tumor development is a complex pathological process, with malignant tumors characterized by excessive cell proliferation and migration. Tumor cells migrate and proliferate in response to signals from stromal cells, immune cells, and vascular cells, promoting further colonization and metastasis (40). To confirm that upregulation of the P4HA2 gene plays an important role in the development of colorectal cancer, the authors examined the expression of P4HA2 in the human normal intestinal epithelial cell line HIEC and the colorectal cancer cell lines LoVo, SW480, SW620, HT29, and HCT8. HCT8 colorectal cancer cells with high P4HA2 expression were selected for gene knockdown, SW480 cells with low P4HA2 expression were selected for P4HA2 overexpression, and stably transfected cell lines with shP4HA2 interference and P4HA2 overexpression were constructed. The results of the positive and negative bidirectional functional experiments, such as the CCK8, Transwell, and scratch assays, suggest that P4HA2 promotes colorectal cancer cell proliferation and migration *in vitro* and that it may have biological effects on colorectal cancer regulation. The results showed that P4HA2 promotes the development of colorectal cancer, but the specific mechanism of P4HA2-mediated colorectal cancer development has not been elucidated.

In previous studies, P4HA2 altered the tumor microenvironment by influencing collagen structure, function, and stability (10, 41). This remodeling can regulate tumor cell polarity, metabolism, and signal transduction (10). P4HA2 can also regulate the expression of tumor-related genes, increase the malignant phenotype of tumor cells, and promote drug resistance (14, 42). P4HA2 dysregulation contributes significantly to tumor growth, invasion, and therapeutic resistance (36). However, more research is needed to determine the precise functions and mechanisms of these central genes in the occurrence and progression of colorectal cancer.

To determine the mechanism of P4HA2 in colorectal cancer, 4D label-free quantitative proteomics was used to identify differentially expressed genes following P4HA2 knockdown. The downregulation of STAT1 expression by P4HA2 was confirmed via qRT-PCR and Western blotting. Studies have demonstrated that P4HA2 governs the expression of STAT1, which, as a crucial molecule of signal transduction within the tumor microenvironment, is intimately associated with the inflammatory response and tumor immune surveillance (17). Increasing evidence indicates that the IFN-γ/JAK2/STAT1 pathway is widely recognized as the principal inducer of PD-L1 expression (43).

High expression of PD-L1 in tumor cells is associated with immune tolerance and drug resistance to anti-PD-L1 therapy. The downregulation of PD-L1 expression in the majority of patients can effectively stimulate the antitumor immune response to enhance immunotherapy (44, 45). P4HA2 not only regulates the formation of the tumor microenvironment but also potentially plays a role in inflammation and tumor immunity. Studies have revealed that P4HA2 upregulates the expression of PD-L1 in cervical cancer tissues and is negatively correlated with CD8+ T cells, regulating antitumor immunity and serving as a prognostic marker for immunotherapy (46). Additionally, P4HA2 is correlated with eight immune checkpoint molecules in colorectal adenocarcinoma, including PD-L1 (47), and all the above studies suggest that the regulation of PD-L1 expression by P4HA2 is related to immunotherapy. Further experiments at the cellular level measured the expression of JAK2, IFN, IRF, and PD-L1 proinflammatory signaling pathway regulators and revealed that P4HA2 upregulated the expression of JAK2, IFN, IRF, and PD-L1, indicating that the upregulation of PD-L1 expression by P4HA2 inhibits antitumor immunity.

In conclusion, P4HA2 may promote the development of colorectal cancer by mediating immune regulation, but this study has several limitations, and the deeper mechanisms of immune regulation require further experimental investigation.

## Conclusions

P4HA2 may affect the STAT1/PD-L1 pathway to promote the development and inhibit antitumor immunity of colorectal cancer by binding to and downregulating the expression of STAT1, which may reveal a new pathogenic mechanism. This highlights its potential as a precision therapy biomarker.

## Data Availability

The datasets presented in this study can be found in online repositories. The names of the repository/repositories and accession number(s) can be found in the article/**Supplementary Material**.

## Supplementary material

Supplementary Figure 1
**(A)** Survival analysis of top 5 upregulated and downregulated proteins from proteomics data in CRC patients using Kaplan-Meier method. **(B)** qPCR validation of proteomics array results showing no significant change in IQGAP2 levels following P4HA2 treatment.
