# Supplementary figures and images for: Integrated analysis identifies P4HA2 as a key regulator of STAT1-mediated colorectal cancer progression and a potential biomarker for precision therapy
Source: Front Oncol. 2025 May 8;15:1581860. doi: 10.3389/fonc.2025.1581860 (PMC12094996; doi:10.3389/fonc.2025.1581860)

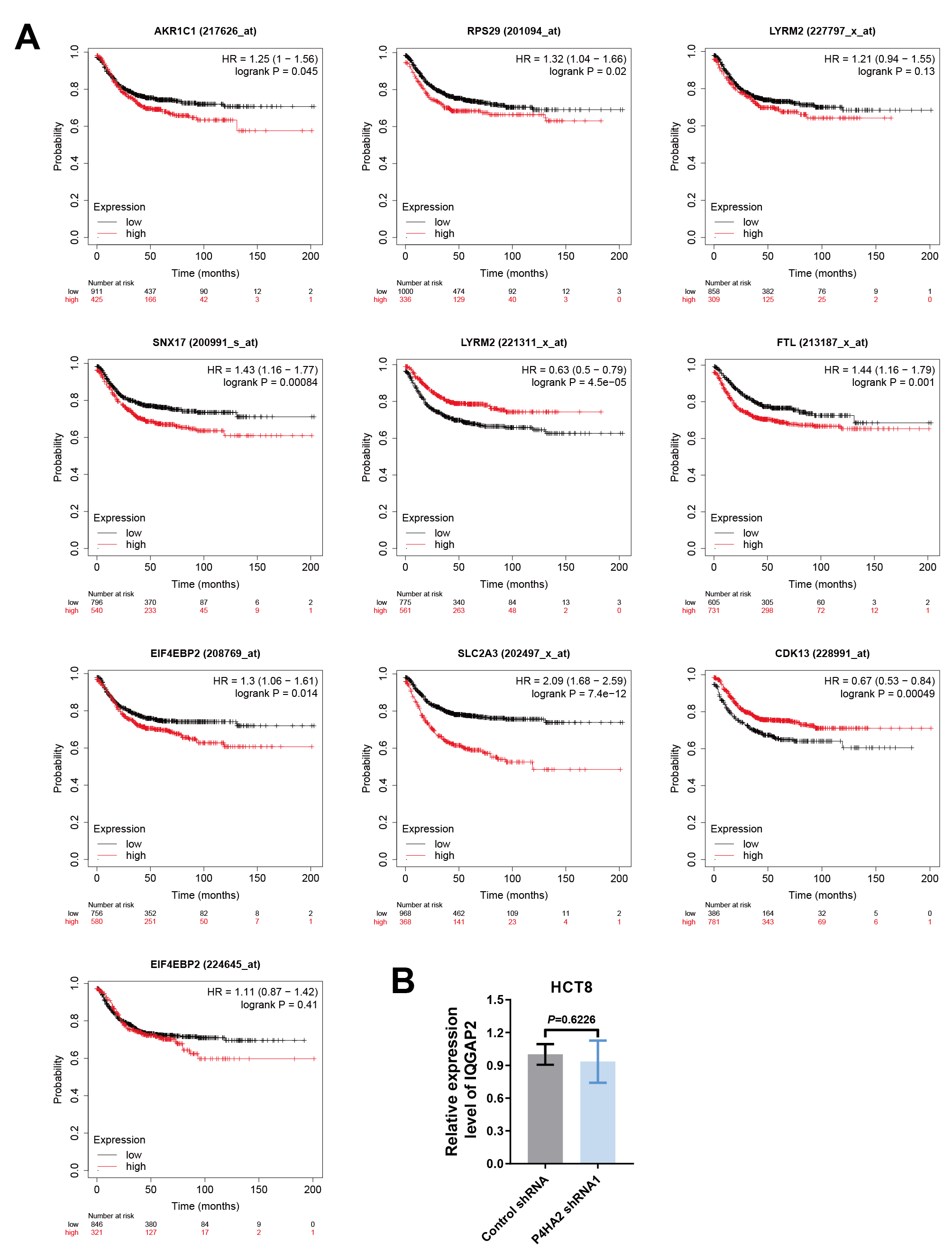

Supplement: Supplementary Figure 1 — (A) Survival analysis of top 5 upregulated and downregulated proteins from proteomics data in CRC patients using Kaplan-Meier method. (B) qPCR validation of proteomics array results showing no significant change in IQGAP2 levels following P4HA2 treatment. [file Image1.jpg]
