# Supplementary material for: Integrated analysis identifies P4HA2 as a key regulator of STAT1-mediated colorectal cancer progression and a potential biomarker for precision therapy
Source: Front Oncol. 2025 May 8;15:1581860. doi: 10.3389/fonc.2025.1581860 (PMC12094996; doi:10.3389/fonc.2025.1581860)
Supplement: Supplementary file 2 [file Table1.docx]

**Supplementary file 1: Table S1. Target sequences for P4HA2 knockdown and overexpression, for STAT1 siRNA and overexpression**

| Name | Sequences |
| --- | --- |
| shP4HA2-1 | 5′-GCAGTCTCTGAAAGAGTACAT-3′ |
| shP4HA2-2 | 5′-CGAGATACTTTCAAGCATTTA-3′ |
| sh-NC | 5′-GTTCTCCGAACGTGTCACTTT-3′ |
| overexpression P4HA2 | NM_004199.3 |
| overexpression STAT1 | NM_007315.4 |
| si-STAT1 sense | 5′- CUCAUUCCGUGGACGAGGUdTdT-3′ |
| si-STAT1 antisense | 5′- ACCUCGUCCACGGAAUGAGdTdT-3′ |
| si-NC sense | 5′- UUCUCCGAACGUGUCACGUdTdT-3′ |
| si-NC antisense | 5′- ACGUGACACGUUCGGAGAAdTdT-3′ |
