# Supplementary material for: Integrated analysis identifies P4HA2 as a key regulator of STAT1-mediated colorectal cancer progression and a potential biomarker for precision therapy
Source: Front Oncol. 2025 May 8;15:1581860. doi: 10.3389/fonc.2025.1581860 (PMC12094996; doi:10.3389/fonc.2025.1581860)
Supplement: Supplementary file 3 [file Table2.doc]

**Supplementary file 2: Table S2. The names of the genes and sequences of primers.**

| **Gene** | **primer sequences** |
| --- | --- |
| P4HA2 | F: 5'-GGCCTGGTTTGGTGTCCTG-3' |
|  | R: 5'-GCCCAGCTCTTAATCTTGGAAAG-3' |
| STAT1 | F: 5'-TGGATCAGCTGCAGAACTGG-3' |
|  | R: 5'-GAAGGTGCGGTCCCATAACA-3' |
| IQGAP2 | F: 5′-TTAGAAACGCAGCAGACAGC-3′ |
|  | R: 5′-CAGAACCAAGGCCGATCACC-3′ |
| IFN-γ | F: 5′-GGCAAGGCTATGTGATTACAAGG-3′ |
|  | R: 5′-CATCAAGTGAAATAAACACACAACCC-3′ |
| JAK2 | F: 5′-TTGTGGTATTACGCCTGTGTATC-3′  R: ­­5′-ATGCCTGGTTGACTCGTCTAT-3′ |
| IRF1 | F: 5′-CATGGCTGGGACATCAACAA-3′  R: ­­5′-TTGTATCGGCCTGTGTGAATG-3′ |
| PD-L1 | F: 5′- TGGCATTTGCTGAACGCATTT -3′  R: ­­5′- TGCAGCCAGGTCTAATTGTTTT -3′ |
| GAPDH | F: 5′-CCTCAAGATCATCAGCAAT-3′ |
|  | R: 5′-CCATCCACAGTCTTCTGGGT-3′ |
