# Supplementary material for: Integrated analysis identifies P4HA2 as a key regulator of STAT1-mediated colorectal cancer progression and a potential biomarker for precision therapy
Source: Front Oncol. 2025 May 8;15:1581860. doi: 10.3389/fonc.2025.1581860 (PMC12094996; doi:10.3389/fonc.2025.1581860)
Supplement: Supplementary file 4 [file Table3.docx]

**Supplementary file 3: Table S3.** **Expression of differential genes.**

| **Gene name** | **logFC** | **AveExpr** | **adj.P.Val** | **Regulated trend** |
| --- | --- | --- | --- | --- |
| IBSP | 1.251321705 | 3.023423737 | 0.004976232 | upregulated |
| C11orf41 | 1.116178564 | 3.979860201 | 0.011703435 | upregulated |
| MDGA1 | 0.969734103 | 5.507549228 | 0.001515575 | upregulated |
| MARCO | 0.954992933 | 7.546768913 | 0.004285166 | upregulated |
| VTCN1 | 0.941364242 | 1.986748013 | 0.025167102 | upregulated |
| COL10A1 | 0.905386275 | 10.9482119 | 0.014323086 | upregulated |
| PCDHB2 | 0.818630903 | 4.246308634 | 0.028938977 | upregulated |
| P4HA2 | 0.801761855 | 12.49963284 | 1.87E-36 | upregulated |
| NPR3 | 0.801625824 | 4.546820379 | 0.016626965 | upregulated |
| CHST6 | 0.78448798 | 7.904845491 | 0.000531306 | upregulated |
| ABCC9 | 0.773498516 | 1.492061311 | 0.010975578 | upregulated |
| COL5A3 | 0.765083631 | 7.115510975 | 0.004518415 | upregulated |
| AVPR1A | 0.760437342 | 2.547081575 | 0.033943915 | upregulated |
| PPFIA4 | 0.730022249 | 4.634942899 | 0.01068563 | upregulated |
| COL7A1 | 0.727312373 | 9.551681107 | 0.006796767 | upregulated |
| HS3ST2 | 0.721581138 | 3.811525181 | 0.019183703 | upregulated |
| RAB3B | 0.718902363 | 5.652726852 | 0.001460228 | upregulated |
| RGS17 | 0.716669986 | 4.092467168 | 0.016699549 | upregulated |
| GLIS3 | 0.69229827 | 3.267046543 | 0.00334108 | upregulated |
| HS3ST3A1 | 0.678450815 | 5.073135093 | 0.030573357 | upregulated |
| UNC5B | 0.678041073 | 11.21707978 | 2.67E-05 | upregulated |
| LOX | 0.669865369 | 8.312590575 | 0.005355687 | upregulated |
| PCOLCE | 0.66954258 | 9.969068197 | 0.001260299 | upregulated |
| GRID1 | 0.668878483 | 4.781830401 | 0.004481134 | upregulated |
| PNPLA3 | 0.661099364 | 7.452492642 | 0.046175983 | upregulated |
| SPOCD1 | 0.660109397 | 8.426553996 | 0.000977347 | upregulated |
| PODNL1 | 0.628282448 | 6.951540488 | 0.003872107 | upregulated |
| AEBP1 | 0.62442988 | 12.1118059 | 0.018467756 | upregulated |
| VEPH1 | 0.619487662 | 4.838894776 | 0.011642769 | upregulated |
| CYTL1 | 0.613034094 | 6.241163678 | 0.023870528 | upregulated |
| FBLN2 | 0.602590057 | 9.36478881 | 0.035913958 | upregulated |
| TIE1 | 0.599680602 | 6.99671338 | 0.009584792 | upregulated |
| MAGED4B | 0.599523941 | 7.97839701 | 0.003872107 | upregulated |
| PAEP | 0.596573366 | 3.351704015 | 0.042588842 | upregulated |
| ADAMTSL4 | 0.590363775 | 3.681861421 | 0.011473657 | upregulated |
| ERMN | 0.589919153 | 3.694432363 | 0.030110746 | upregulated |
| CCDC36 | 0.583881554 | 4.050921054 | 0.018949276 | upregulated |
| GCNT4 | 0.582857309 | 2.63483714 | 0.014272542 | upregulated |
| ALPK2 | 0.580567177 | 5.582519065 | 0.024909101 | upregulated |
| ADAMTS4 | 0.572897716 | 11.14188882 | 0.038050185 | upregulated |
| HTRA3 | 0.569691364 | 5.744001292 | 0.02471737 | upregulated |
| NETO2 | 0.56207197 | 9.825279078 | 0.029329769 | upregulated |
| SPON2 | 0.558042783 | 14.79733929 | 0.02805083 | upregulated |
| LRRC4C | 0.557866954 | 3.179063622 | 0.045639836 | upregulated |
| BAIAP3 | 0.55761657 | 4.897709129 | 0.032953548 | upregulated |
| CHSY3 | 0.554039014 | 5.405314866 | 0.010565349 | upregulated |
| AR | 0.548815191 | 5.990899847 | 0.029439318 | upregulated |
| SLC4A3 | 0.547011362 | 5.943403 | 0.040172255 | upregulated |
| TSPAN9 | 0.546872948 | 7.072586795 | 0.002401979 | upregulated |
| ARSI | 0.545233434 | 5.474369816 | 0.007824143 | upregulated |
| ALOX5 | 0.543972456 | 11.45775797 | 0.001115779 | upregulated |
| MLC1 | 0.543290089 | 5.369605212 | 0.020545773 | upregulated |
| BGN | 0.54236286 | 7.622786156 | 0.004797203 | upregulated |
| SLC16A3 | 0.541864694 | 14.56186808 | 0.000442874 | upregulated |
| DBN1 | 0.541073233 | 11.34781004 | 0.002381985 | upregulated |
| LOXL2 | 0.536702364 | 11.92846753 | 0.028035421 | upregulated |
| COL6A2 | 0.53075192 | 9.030643966 | 0.013510712 | upregulated |
| PLVAP | 0.529615452 | 9.214956871 | 0.012556013 | upregulated |
| COL5A2 | 0.526384356 | 12.98509091 | 0.043250621 | upregulated |
| ECM1 | 0.5173908 | 10.48725972 | 0.002220038 | upregulated |
| HMOX1 | 0.509938259 | 11.52486257 | 0.014624143 | upregulated |
| AMIGO2 | 0.50129629 | 9.80024098 | 0.024617576 | upregulated |
| GSTO2 | -0.517833865 | 9.851731867 | 0.01068563 | downregulated |
| CTTNBP2 | -0.525750952 | 9.250598976 | 0.017323708 | downregulated |
| C10orf116 | -0.526207344 | 14.11107914 | 0.038823515 | downregulated |
| APOM | -0.531976064 | 9.302411809 | 0.001519637 | downregulated |
| STOX1 | -0.532743239 | 7.760759098 | 0.030655926 | downregulated |
| LRRC2 | -0.539744057 | 8.138269975 | 0.008461367 | downregulated |
| P2RY12 | -0.544126058 | 3.395922792 | 0.023069955 | downregulated |
| SPACA3 | -0.548596643 | 8.156040569 | 0.007606369 | downregulated |
| BTBD16 | -0.549659279 | 8.43883305 | 0.023491665 | downregulated |
| GZMB | -0.559110575 | 11.92572247 | 0.037214299 | downregulated |
| TINAG | -0.580340101 | 6.85233943 | 0.015715494 | downregulated |
| IDI2 | -0.600894749 | 3.179937271 | 0.021600772 | downregulated |
| AMBP | -0.627529856 | 5.288568303 | 0.047667608 | downregulated |
| CLECL1 | -0.635359609 | 6.027648416 | 0.018467756 | downregulated |
| UNC5D | -0.647922854 | 1.215978362 | 0.021529993 | downregulated |
| IFNG | -0.648953653 | 4.885499884 | 0.025807902 | downregulated |
| RLN1 | -0.66341909 | 5.10169879 | 0.012283552 | downregulated |
| SLC27A5 | -0.682094494 | 11.22549726 | 0.023197322 | downregulated |
| RPS6KA6 | -0.696185432 | 5.705908186 | 0.008889343 | downregulated |
| HLA-DQB1 | -0.723294214 | 11.51699274 | 0.015743722 | downregulated |
| RLN2 | -0.75482132 | 6.515494768 | 0.006710794 | downregulated |
| ZNF541 | -0.838519238 | 6.652746845 | 0.010488291 | downregulated |
| TNMD | -0.901836055 | 5.10383788 | 0.023870528 | downregulated |
| IZUMO2 | -1.049648785 | 7.351510731 | 0.002345829 | downregulated |
