# Supplementary material for: Integrated analysis identifies P4HA2 as a key regulator of STAT1-mediated colorectal cancer progression and a potential biomarker for precision therapy
Source: Front Oncol. 2025 May 8;15:1581860. doi: 10.3389/fonc.2025.1581860 (PMC12094996; doi:10.3389/fonc.2025.1581860)
Supplement: Supplementary file 5 [file Table4.docx]

**Supplementary file 4: Table S4. Differentially expressed proteins regulation by P4HA2 in HCT8 cells.**

| **Protein accession** | **Gene name** | **sh1/shNC Ratio** | **sh1/shNC P value** | **Regulated Type** |
| --- | --- | --- | --- | --- |
| P11169 | SLC2A3 | 5.643 | 7.98E-05 | Up |
| P02792 | FTL | 5.325 | 0.038178 | Up |
| Q04828 | AKR1C1 | 5.226 | 0.005952 | Up |
| Q13576 | IQGAP2 | 4.899 | 0.000295 | Up |
| P42224 | STAT1 | 4.504 | 1.5E-06 | Up |
| Q9P2M4 | TBC1D14 | 3.837 | 0.000645 | Up |
| P13688 | CEACAM1 | 3.429 | 0.000315 | Up |
| P13196 | ALAS1 | 3.341 | 0.0039 | Up |
| Q14571 | ITPR2 | 3.283 | 0.009692 | Up |
| Q96IW7 | SEC22A | 2.967 | 0.000958 | Up |
| P16444 | DPEP1 | 2.885 | 2.05E-05 | Up |
| L0R8F8 | MIEF1 | 2.82 | 5.48E-05 | Up |
| O76070 | SNCG | 2.775 | 6.99E-05 | Up |
| Q12982 | BNIP2 | 2.675 | 0.000885 | Up |
| Q66K89 | E4F1 | 2.67 | 0.002287 | Up |
| Q9BV73 | CEP250 | 2.564 | 0.00212 | Up |
| Q9UII4 | HERC5 | 2.557 | 0.003605 | Up |
| P21980 | TGM2 | 2.525 | 0.012281 | Up |
| Q9BRQ6 | CHCHD6 | 2.461 | 0.016778 | Up |
| Q9NYL4 | FKBP11 | 2.343 | 0.000341 | Up |
| Q9Y289 | SLC5A6 | 2.298 | 0.044546 | Up |
| Q9UHN1 | POLG2 | 2.237 | 0.015692 | Up |
| P62166 | NCS1 | 2.227 | 0.032764 | Up |
| P22830 | FECH | 2.171 | 3.09E-05 | Up |
| Q06203 | PPAT | 2.155 | 7.25E-05 | Up |
| Q9H9Y2 | RPF1 | 2.08 | 0.028121 | Up |
| P35908 | KRT2 | 2.056 | 0.01021 | Up |
| Q9Y5B0 | CTDP1 | 2.04 | 3.76E-05 | Up |
| Q12913 | PTPRJ | 2.026 | 8.47E-05 | Up |
| P21912 | SDHB | 2.021 | 3.41E-05 | Up |
| P09923 | ALPI | 2.016 | 0.000337 | Up |
| Q14145 | KEAP1 | 1.953 | 0.0007 | Up |
| P78345 | RPP38 | 1.939 | 0.022128 | Up |
| L0R6Q1 | SLC35A4 | 1.907 | 0.000758 | Up |
| P02794 | FTH1 | 1.898 | 1.41E-05 | Up |
| Q9UK59 | DBR1 | 1.891 | 0.001179 | Up |
| P51798 | CLCN7 | 1.842 | 0.018502 | Up |
| P00973 | OAS1 | 1.823 | 0.009541 | Up |
| Q9HBI6 | CYP4F11 | 1.818 | 0.032206 | Up |
| Q14315 | FLNC | 1.812 | 4.27E-06 | Up |
| P09914 | IFIT1 | 1.791 | 0.001872 | Up |
| P18074 | ERCC2 | 1.771 | 0.004121 | Up |
| Q96S82 | UBL7 | 1.759 | 0.001085 | Up |
| Q6NXR4 | TTI2 | 1.726 | 0.002508 | Up |
| P15328 | FOLR1 | 1.722 | 0.000327 | Up |
| P78563 | ADARB1 | 1.716 | 0.012177 | Up |
| Q99541 | PLIN2 | 1.698 | 0.006841 | Up |
| Q92934 | BAD | 1.698 | 0.023129 | Up |
| Q86UT6 | NLRX1 | 1.684 | 0.000105 | Up |
| O94829 | IPO13 | 1.678 | 0.022667 | Up |
| O00625 | PIR | 1.675 | 0.020029 | Up |
| Q9NP58 | ABCB6 | 1.674 | 0.004841 | Up |
| Q6ZMK1 | CYHR1 | 1.672 | 0.046177 | Up |
| Q96MY1 | NOL4L | 1.672 | 0.042896 | Up |
| Q9NZD8 | SPG21 | 1.671 | 0.012139 | Up |
| P42338 | PIK3CB | 1.663 | 0.000436 | Up |
| P62745 | RHOB | 1.663 | 0.002223 | Up |
| O14879 | IFIT3 | 1.661 | 0.005219 | Up |
| Q8IYB7 | DIS3L2 | 1.639 | 0.02752 | Up |
| Q4G0J3 | LARP7 | 1.624 | 0.000157 | Up |
| Q99519 | NEU1 | 1.606 | 0.012118 | Up |
| Q07020 | RPL18 | 1.592 | 0.005208 | Up |
| O76021 | RSL1D1 | 1.586 | 0.000276 | Up |
| Q8IYB8 | SUPV3L1 | 1.585 | 0.028228 | Up |
| Q86UY6 | NAA40 | 1.582 | 0.037431 | Up |
| Q15646 | OASL | 1.582 | 0.000114 | Up |
| O95456 | PSMG1 | 1.575 | 0.048578 | Up |
| Q9BTA9 | WAC | 1.573 | 0.000105 | Up |
| Q15147 | PLCB4 | 1.567 | 0.00118 | Up |
| Q969S9 | GFM2 | 1.56 | 0.001755 | Up |
| Q96DU7 | ITPKC | 1.553 | 0.004196 | Up |
| P20393 | NR1D1 | 1.549 | 0.010044 | Up |
| Q9BYX2 | TBC1D2 | 1.548 | 0.049613 | Up |
| Q13530 | SERINC3 | 1.547 | 0.012079 | Up |
| Q14534 | SQLE | 1.546 | 0.015253 | Up |
| Q96MX6 | DNAAF10 | 1.538 | 0.015116 | Up |
| Q6UWU4 | C6orf89 | 1.534 | 0.002968 | Up |
| Q8WTT2 | NOC3L | 1.527 | 0.000217 | Up |
| Q99643 | SDHC | 1.527 | 0.002581 | Up |
| Q9P2R6 | RERE | 1.526 | 0.029616 | Up |
| Q9H019 | MTFR1L | 1.519 | 0.04785 | Up |
| P32780 | GTF2H1 | 1.519 | 3.65E-05 | Up |
| P80404 | ABAT | 1.517 | 0.011648 | Up |
| O94901 | SUN1 | 1.507 | 0.001773 | Up |
| Q6PCE3 | PGM2L1 | 1.505 | 0.022942 | Up |
| Q9NZW5 | PALS2 | 1.503 | 0.000274 | Up |
| Q13557 | CAMK2D | 1.503 | 0.042496 | Up |
| O43920 | NDUFS5 | 1.503 | 0.009648 | Up |
| Q14004 | CDK13 | 0.142 | 7.15E-05 | Down |
| Q15036 | SNX17 | 0.147 | 9.55E-05 | Down |
| Q13542 | EIF4EBP2 | 0.164 | 7.22E-05 | Down |
| Q9NU23 | LYRM2 | 0.2 | 0.004436 | Down |
| P62273 | RPS29 | 0.259 | 2.41E-05 | Down |
| Q9H7X7 | IFT22 | 0.29 | 0.000886 | Down |
| Q9H4H8 | FAM83D | 0.328 | 0.026175 | Down |
| Q01804 | OTUD4 | 0.37 | 0.008027 | Down |
| Q6IBW4 | NCAPH2 | 0.378 | 6.66E-05 | Down |
| Q9BRD0 | BUD13 | 0.383 | 0.014382 | Down |
| Q9BUB5 | MKNK1 | 0.389 | 0.00015 | Down |
| Q9Y6X5 | ENPP4 | 0.398 | 0.000149 | Down |
| Q9BY49 | PECR | 0.402 | 0.002431 | Down |
| P21589 | NT5E | 0.409 | 0.005955 | Down |
| Q96H79 | ZC3HAV1L | 0.418 | 0.017287 | Down |
| Q3KQU3 | MAP7D1 | 0.426 | 0.009439 | Down |
| Q53HL2 | CDCA8 | 0.428 | 0.042491 | Down |
| P31321 | PRKAR1B | 0.431 | 0.028482 | Down |
| Q6IA17 | SIGIRR | 0.434 | 3.84E-05 | Down |
| Q9NZD2 | GLTP | 0.443 | 0.015951 | Down |
| P23511 | NFYA | 0.445 | 0.000116 | Down |
| Q9Y4I1 | MYO5A | 0.456 | 0.03359 | Down |
| Q5T0W9 | FAM83B | 0.465 | 0.000545 | Down |
| Q9Y448 | KNSTRN | 0.469 | 0.010478 | Down |
| P63173 | RPL38 | 0.476 | 0.000596 | Down |
| O43189 | PHF1 | 0.487 | 0.001754 | Down |
| Q9Y572 | RIPK3 | 0.492 | 0.017922 | Down |
| P28065 | PSMB9 | 0.492 | 0.030115 | Down |
| Q96EX3 | DYNC2I2 | 0.494 | 0.005051 | Down |
| P29966 | MARCKS | 0.502 | 1.24E-05 | Down |
| O43572 | AKAP10 | 0.504 | 0.030161 | Down |
| P51692 | STAT5B | 0.508 | 0.002497 | Down |
| Q9H2F3 | HSD3B7 | 0.51 | 0.011266 | Down |
| Q7LGA3 | HS2ST1 | 0.531 | 0.001617 | Down |
| Q9HCH5 | SYTL2 | 0.538 | 0.037989 | Down |
| P83731 | RPL24 | 0.539 | 0.005383 | Down |
| Q07864 | POLE | 0.549 | 0.042241 | Down |
| Q4G148 | GXYLT1 | 0.552 | 0.037662 | Down |
| Q12894 | IFRD2 | 0.553 | 0.034001 | Down |
| O00628 | PEX7 | 0.556 | 0.012151 | Down |
| P48436 | SOX9 | 0.574 | 0.00014 | Down |
| O94887 | FARP2 | 0.578 | 0.006277 | Down |
| P67809 | YBX1 | 0.579 | 0.00028 | Down |
| O15055 | PER2 | 0.586 | 0.011108 | Down |
| Q14011 | CIRBP | 0.586 | 0.002086 | Down |
| Q9NZM1 | MYOF | 0.594 | 1.22E-05 | Down |
| Q9H8V3 | ECT2 | 0.595 | 0.004321 | Down |
| Q8NC96 | NECAP1 | 0.598 | 0.005911 | Down |
| Q9H3R2 | MUC13 | 0.602 | 0.001946 | Down |
| Q96N66 | MBOAT7 | 0.602 | 0.00081 | Down |
| O00193 | SMAP | 0.603 | 0.003774 | Down |
| Q969R2 | OSBP2 | 0.604 | 0.038082 | Down |
| P18827 | SDC1 | 0.605 | 0.027656 | Down |
| Q8NEM2 | SHCBP1 | 0.606 | 0.000908 | Down |
| P31350 | RRM2 | 0.606 | 2.57E-05 | Down |
| Q9BRV3 | SLC50A1 | 0.609 | 0.00036 | Down |
| Q9NQW6 | ANLN | 0.611 | 0.000517 | Down |
| O15121 | DEGS1 | 0.611 | 0.002208 | Down |
| Q6MZP7 | LIN54 | 0.612 | 0.041935 | Down |
| Q86WP2 | GPBP1 | 0.612 | 0.010364 | Down |
| Q6YHU6 | THADA | 0.614 | 0.021943 | Down |
| Q9H3G5 | CPVL | 0.615 | 0.010996 | Down |
| P30260 | CDC27 | 0.617 | 0.001482 | Down |
| Q96R06 | SPAG5 | 0.619 | 0.000667 | Down |
| Q8TBP6 | SLC25A40 | 0.62 | 0.0002 | Down |
| Q9H9E3 | COG4 | 0.62 | 0.048158 | Down |
| O94806 | PRKD3 | 0.621 | 0.003438 | Down |
| Q9H0W8 | SMG9 | 0.622 | 0.024353 | Down |
| Q8TF72 | SHROOM3 | 0.623 | 0.010552 | Down |
| Q9UM54 | MYO6 | 0.623 | 0.002235 | Down |
| Q96B23 | C18orf25 | 0.624 | 0.006987 | Down |
| O60869 | EDF1 | 0.627 | 2.85E-05 | Down |
| Q9NP61 | ARFGAP3 | 0.63 | 0.00496 | Down |
| P62899 | RPL31 | 0.631 | 0.015813 | Down |
| Q04760 | GLO1 | 0.633 | 1.95E-05 | Down |
| Q8WUA2 | PPIL4 | 0.639 | 0.000195 | Down |
| P50897 | PPT1 | 0.639 | 0.040037 | Down |
| Q8WVX9 | FAR1 | 0.639 | 0.006062 | Down |
| Q9BV57 | ADI1 | 0.64 | 0.00899 | Down |
| Q16890 | TPD52L1 | 0.64 | 5.38E-06 | Down |
| P62841 | RPS15 | 0.641 | 0.002002 | Down |
| Q9BV44 | THUMPD3 | 0.641 | 0.001424 | Down |
| Q5T8D3 | ACBD5 | 0.642 | 0.000212 | Down |
| O75496 | GMNN | 0.643 | 0.006342 | Down |
| Q99547 | MPHOSPH6 | 0.647 | 0.046654 | Down |
| Q5T5C0 | STXBP5 | 0.648 | 0.013474 | Down |
| A6NKF1 | SAC3D1 | 0.649 | 0.039583 | Down |
| P51808 | DYNLT3 | 0.65 | 0.001955 | Down |
| O75362 | ZNF217 | 0.65 | 0.049436 | Down |
| Q8NFW8 | CMAS | 0.65 | 0.043095 | Down |
| Q6IAA8 | LAMTOR1 | 0.652 | 0.01009 | Down |
| P08581 | MET | 0.654 | 0.001798 | Down |
| Q14244 | MAP7 | 0.655 | 0.005266 | Down |
| Q6IQ49 | SDE2 | 0.656 | 0.008239 | Down |
| Q96FQ6 | S100A16 | 0.656 | 0.000235 | Down |
| Q9BWT6 | MND1 | 0.656 | 0.005782 | Down |
| P61353 | RPL27 | 0.656 | 0.000565 | Down |
| P48634 | PRRC2A | 0.657 | 0.000467 | Down |
| Q9H098 | FAM107B | 0.657 | 0.014104 | Down |
| Q6UX07 | DHRS13 | 0.66 | 0.001107 | Down |
| Q14185 | DOCK1 | 0.66 | 0.012344 | Down |
| Q6PHR2 | ULK3 | 0.661 | 0.010829 | Down |
| P46783 | RPS10 | 0.662 | 0.00075 | Down |
| P60520 | GABARAPL2 | 0.663 | 0.026263 | Down |
| Q8N1F8 | STK11IP | 0.664 | 0.028133 | Down |
| Q93052 | LPP | 0.664 | 0.004903 | Down |
